# Investigating the genetic basis of maize ear characteristics: a comprehensive genome-wide study utilizing high-throughput phenotypic measurement method and system

**DOI:** 10.3389/fpls.2023.1248446

**Published:** 2023-08-28

**Authors:** Jinglu Wang, Shuaihao Zhao, Ying Zhang, Xianju Lu, Jianjun Du, Chuanyu Wang, Weiliang Wen, Xinyu Guo, Chunjiang Zhao

**Affiliations:** ^1^ College of Plant Science and Technology, Huazhong Agricultural University, Wuhan, China; ^2^ National Engineering Research Center for Information Technology in Agriculture, Beijing Academy of Agriculture and Forestry Sciences, Beijing, China; ^3^ Beijing Key Lab of Digital Plant, Information Technology Research Center, Beijing Academy of Agriculture and Forestry Sciences, Beijing, China

**Keywords:** maize, high-throughput phenotypic measurement, ear-related traits, color traits, GWAS, candidate genes

## Abstract

The morphology of maize ears plays a critical role in the breeding of new varieties and increasing yield. However, the study of traditional ear-related traits alone can no longer meet the requirements of breeding. In this study, 20 ear-related traits, including size, shape, number, and color, were obtained in 407 maize inbred lines at two sites using a high-throughput phenotypic measurement method and system. Significant correlations were found among these traits, particularly the novel trait ear shape (ES), which was correlated with traditional traits: kernel number per row and kernel number per ear. Pairwise comparison tests revealed that the inbred lines of tropical-subtropical were significantly different from other subpopulations in row numbers per ear, kernel numbers per ear, and ear color. A genome-wide association study identified 275, 434, and 362 Single nucleotide polymorphisms (SNPs) for Beijing, Sanya, and best linear unbiased prediction scenarios, respectively, explaining 3.78% to 24.17% of the phenotypic variance. Furthermore, 58 candidate genes with detailed functional descriptions common to more than two scenarios were discovered, with 40 genes being associated with color traits on chromosome 1. After analysis of haplotypes, gene expression, and annotated information, several candidate genes with high reliability were identified, including *Zm00001d051328* for ear perimeter and width, *zma-MIR159f* for ear shape, *Zm00001d053080* for kernel width and row number per ear, and *Zm00001d048373* for the blue color channel of maize kernels in the red-green-blue color model. This study emphasizes the importance of researching novel phenotypic traits in maize by utilizing high-throughput phenotypic measurements. The identified genetic loci enrich the existing genetic studies related to maize ears.

## 1 Introduction

Maize (*Zea mays* L.) has become one of the paramount staple crops worldwide, surpassing the production of wheat or rice. Maize not only is consumed directly but also is extensively used as animal feed and industrial raw material, leading to an increased demand for maize in contemporary society (Gao et al., 2022). However, recent and frequent natural disasters have posed great challenges to food production, including maize. Therefore, ensuring food security and increasing maize yields are the basic objectives of maize production. Among the various methods to increase maize yield, selection and planting of new varieties is one of the most critical technologies. Morphological characteristics of maize ears are key factors in the selection of new varieties. In addition, the maize ear serves as a significant storage reservoir for photosynthetic products in maize and is directly linked to yield. Therefore, it is important to study the phenotype and genetic patterns of maize ears to improve maize yield and quality.

Phenotypic traits of maize ears have been a major concern in maize breeding, especially yield-related traits such as ear size, number of rows, and kernel size. Traditionally, these traits have been derived from manual measurements and visual calculations, which are labor-intensive and time-consuming. However, with the development of computer technology and the application of machine vision and image analysis techniques, fully automated maize variety testing systems have been developed, to capture maize ear-related traits with high-throughput and high accuracy in large-scale breeding programs. For example, Song et al. (2017) developed a high-throughput seed testing device that integrated maize cob testing, threshing, and seed testing into a single device, which significantly improved the efficiency of seed testing. Du et al. (2018) provided a panoramic surface image-based variety test method and system for maize ears assembly line, which achieved a high computational accuracy for various traits. In addition, Liang et al. (2021) designed a high-throughput and low-cost maize ear trait scorer that can be used for ear scoring. All these developments provide the foundation and technical support for high-throughput acquisition of maize ear-related traits and study of maize phenomics, which ultimately contribute to improving maize yield and food security.

An optimized research strategy, genome-wide association study (GWAS), has been widely used to study the relationship between phenotype and genotype at the population level, not only in human diseases but also as a routine tool for gene mining of quantitative traits in crops, such as maize vascular bundle (Zhang et al., 2021; Guo et al., 2022; Sun et al., 2022), plant height (Yang et al., 2014; Farfan et al., 2015; Li et al., 2016), flowering time (Hung et al., 2012; Yang et al., 2013; Yang et al., 2014; Farfan et al., 2015), and husk (Cui et al., 2016; Cui et al., 2020; Jiang et al., 2020). Furthermore, GWAS has also been applied to the study of maize ear-related traits (Xiao et al., 2016; Zhu et al., 2018; Yang et al., 2020; Zhang et al., 2022). For the morphological traits of ears and kernels, Zhou et al. (2018) identified candidate genes for maize ear length (EL) by combining three mapping strategies; Luo et al. (2022) found that genetic variation in *YIGE1* contributed to maize EL and yield. In addition, candidate genes for kernel length and ear diameter were reported (Zhu et al., 2018) to include ubiquitin-activating enzyme (*GRMZM2G015287*), carotenoid cleavage dioxygenase (*GRMZM2G446858*), MYB-CC–type transcription factor, and phosphate starvation response protein 3. For the number of kernel rows, *KRN4* was found to control quantitative variation in maize kernel row number (Liu et al., 2015). Zhang et al. (2022) found that a gene encoding a serine/threonine protein kinase, *Zm00001d016656*, was associated with five yield-related traits in multiple environments. Although there are many studies on ear-related traits, there are few studies on genetic analysis of ear-related traits obtained from high-throughput ear testing systems. Therefore, there is still a great potential to explore the genetic basis of ear-related traits using data obtained from high-throughput ear testing systems. This approach can greatly improve the efficiency and accuracy of identifying potential genes and pathways for maize ear-related traits, which will ultimately help to breed new maize varieties with higher yield and better quality.

In this study, 20 ear-related traits were obtained from 407 inbred lines from the maize association analysis population (Yang et al., 2011) using a high-throughput phenotypic measurement method and system based on panoramic surface image (Du et al., 2018). These traits included maize ear size, shape, number, and color, 13 of which were novel and different from the traditional ear testing traits. These 20 ear-related traits were analyzed by GWAS using Genome-wide efficient mixed model association (GEMMA) (Zhou and Stephens, 2012), which was obtained from two scenarios and best linear unbiased prediction (BLUP) values. The objectives of this study were (1) to analyze the phenotypic characteristics of ear-related traits, with emphasis on the significance of the novel traits, and (2) to identify the key genetic loci and candidate genes for ear-related traits, providing a reference for the genetic basis of the novel ear testing traits.

## 2 Materials and methods

### 2.1 Plant materials and sample collection

The materials used in this study were 407 inbred lines from the maize association mapping panel published by Yang et al. (2011). These lines were classified into four subpopulations: non-stiff stalk (NSS) with 108 lines, stiff stalk (SS) with 31 lines, tropical-subtropical (TST) with 175 lines, and 93 mixed lines (Mixed). The plants were cultivated at two sites in China: in 2019 (SY) at the Nanfan breeding station of Maize Research Center, Beijing Academy of Agriculture and Forestry (Yazhou District, Sanya City, Hainan Province; longitude, 109.1832; latitude, 18.3623) and in 2020 (BJ) at the Beijing Academy of Agriculture and Foresting Science (Tongzhou District, Beijing City; longitude, 116.65; latitude, 39.92). All field trials were conducted in a randomized complete design with planting density and water and fertilizer management based on local field (Lu et al., 2020). At maturity, approximately five uniform ears of each inbred line were selected for phenotypic measurements.

### 2.2 Phenotype acquisition of maize ear

**Figure 1:**
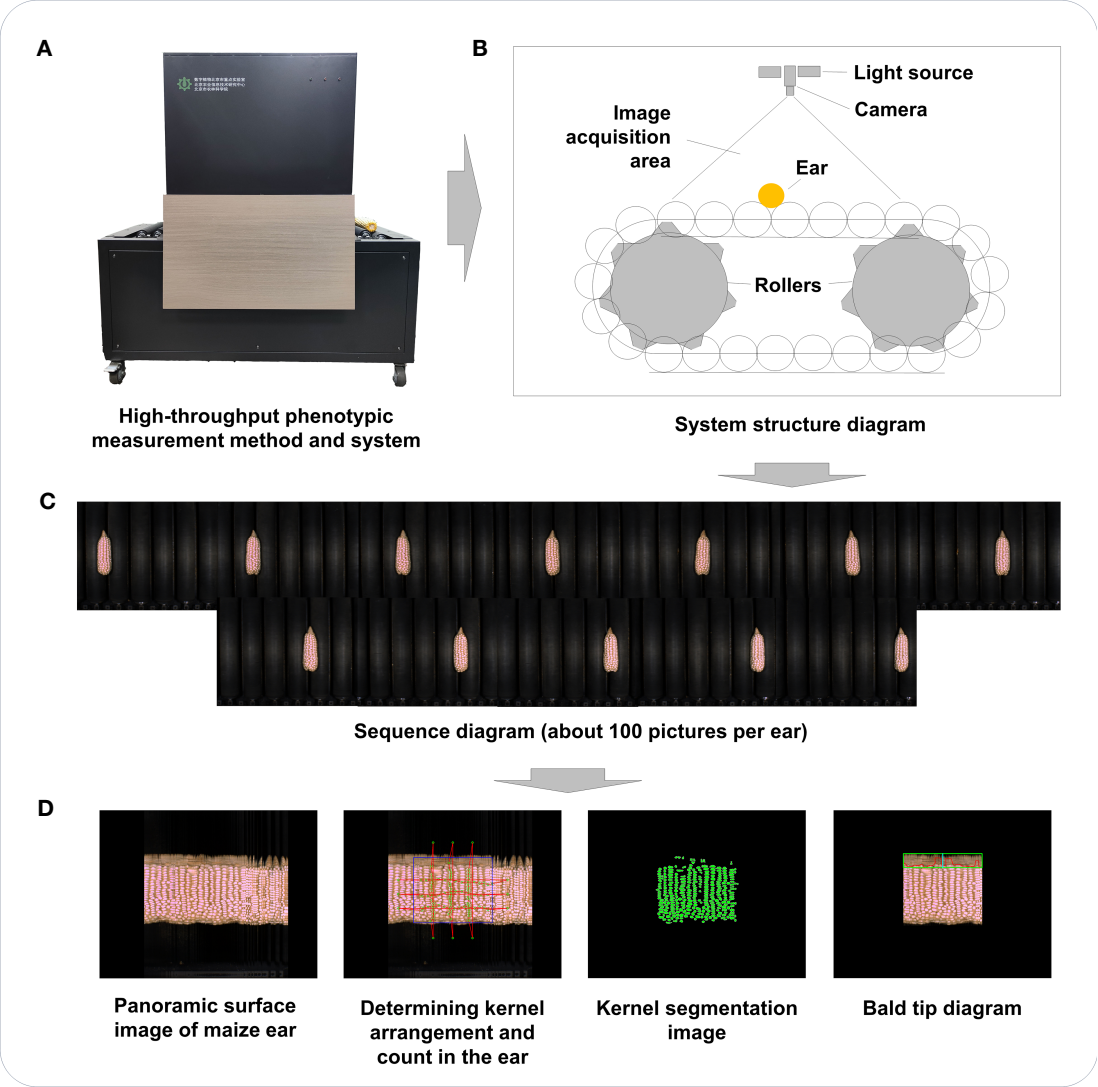
Flowchart of conducting high-throughput phenotypic measurements of maize ear traits. **(A)** The high-throughput phenotypic measurement method and system used in this study. **(B)** System structure diagram. **(C)** Image sequence diagram of maize ear (from left to right, then from top to bottom). **(D)** Panoramic surface image and image segmentation for maize ear.

Maize ear-related phenotype acquisition was carried out by a high-throughput phenotypic measurement method and system (**Figure 1A**) based on panoramic surface image (Du et al., 2018). As shown in **Figure 1**, the system consisted of an imaging unit, computing unit, and ear transmission unit and included a roller conveyor for automatic and continuous transfer of maize ears (**Figure 1B**). The imaging unit was composed of an industrial camera and two light emitting diode (LED) illumination lamps, which detected ear movement and collected real-time images to create an image sequence (**Figure 1C**) covering the entire surface of the ear.

The system had a pallet translation speed of 121 pixels/s, with an imaging unit equipped with two 30-W LED illumination lamps and a high-frame-rate charge coupled device (CCD) industrial camera [MER-125 series, 1/3-inch CCD sensor, 30 frames per second (fps)] with resolution of 1.25 million pixels. The working frame rate for camera image acquisition was approximately 15 fps, and the image was saved in a JPEG format with a resolution of 1,292 × 964 pixels and a pixel size of 3.126 × 10^−2^ cm/pixel. OpenCV was used for image processing, and Visual C++ was used to develop modules for corncob state detection, image acquisition, image stitching, phenotype calculation, and statistical analysis modules (Du et al., 2018).

By analyzing the color differences between the background and the roller chain in ear images, the state of the ear was detected, triggering image acquisition upon placing the ear in the imaging area. Ear segmentation employed color features, and the ear’s shape was enhanced through morphological calculations. It is important to note that the ear image represents a two-dimensional projection of the ear on a plane. On the surface of the ear, the kernels exhibited complex distortions within the image, and the shape and size of the kernels varied significantly across different positions. Comparatively smaller distortions were observed in the small region near the central axis of the ear. Extracting regions with the least distortions from the sequence of ear images allowed for stitching them together to obtain a panoramic image of the ear’s surface. Effective kernels were identified and extracted using ear boundary detection, kernel segmentation, and validity identification techniques. **Figure 1D** shows, notably, that determining the number of rows and kernels per row involved the calculation of centroids for each ear kernel to establish a distribution node reflecting kernel arrangement. Beyond the kernel distribution area, a set of nodes (including a start point and an end point) was defined to represent the direction of kernel arrangement. The Delaunay triangulation method was then applied to connect these nodes, forming a triangular mesh that represented the adjacent relationships between the kernels. The Bellman–Ford method was subsequently utilized to determine the shortest path between each start and end point group, indicating the number of kernels passed through each path and representing the number of rows or kernels per row. Twenty ear-related traits were obtained using this high-throughput phenotypic measurement method and system (**Table 1**).

**Table 1:** Description of 20 traits obtained by high-throughput phenotypic measurement method and system.

Traits	Abbreviations	Descriptions	Unit	Category
Barren tip length	BTL	The projected length from the top of the ear to the furthest kernel on the ear.	cm	Traditional trait
Ear length	EL	Average ear height in each image after axial distortion correction.	cm	Traditional trait
Ear perimeter	EP	Average ear diameter calculated from image sequence using 2πr formula for circumference.	cm	Traditional trait
Ear width	EW	Average value of the diameter of ears.	cm	Traditional trait
Kernel number per ear	KNE	Total number of kernels on each ear of maize.	count	Traditional trait
Kernel number per row	KNR	Kernel count for the central row of maize ear is identified by selecting the highest count of kernels within the central area of the ear image.	count	Traditional trait
Row number per ear	RNE	Divide maize ear image into three paths from left to right, identify the number of kernel rows in each path, calculate the average, and use it as the final row count	count	Traditional trait
Average kernel area	AKA	Projected area of the kernel canopy after the kernels are spread out.	cm^2^	Novel trait
Barren tip area	BTA	The surface area on top of the ear/cob without kernels.	cm^2^	Novel trait
Ear B	EB	The mean blue (B) color channel value in the effective region of a maize ear.	–	Novel trait
Ear G	EG	The mean green (G) color channel value in the effective region of a maize ear.	–	Novel trait
Ear R	ER	The mean red (R) color channel value in the effective region of a maize ear.	–	Novel trait
Ear shape	ES	Uses a non-sphericity index to indicate the similarity of a maize ear’s shape to that of a sphere.	–	Novel trait
Ear surface area	ESA	Calculated using numerical integration to determine the surface area of a maize ear.	cm^2^	Novel trait
Ear volume	EV	Calculated using numerical integration to determine the volume of a maize ear.	cm^3^	Novel trait
Kernel B	KB	Mean value of the B color channel of kernels within the effective area.	–	Novel trait
Kernel G	KG	Mean value of the G color channel of kernels within the effective area.	–	Novel trait
Kernel R	KR	Mean value of the R color channel of kernels within the effective area.	–	Novel trait
Kernel thickness	KT	Average distance between adjacent kernels is considered as kernel thickness to determine distance between centers of adjacent kernels along the row kernel path.	cm	Novel trait
Kernel width	KW	Average length of the crown of the kernel.	cm	Novel trait

### 2.3 Statistical analysis of phenotypic data

Descriptive statistical analysis of phenotypic data distributions was conducted using R (version 3.6.3) software, including the generation of histograms. An analysis of variance (ANOVA) was carried out to assess the differences in traits across subpopulations, utilizing the Holm method (Holm, 1979) to account for multiple testing corrections. In addition, the relationship between traits was evaluated using the Spearman’s rank correlation coefficient method, and, to ensure accurate and robust results, Holm correction (Holm, 1979) was applied for multiple testing.

The BLUP for the 20 phenotypic traits was estimated with the following linear mixed model in ASReml-R v.4.0 by using the “asreml” function of R package asreml (Butler, 2009). The formula for BLUP is


(1) Y=Xβ+Zu+ϵ

where *Y* is the vector of phenotypic values, *X* is the design matrix for fixed effects, *β* is the vector of the coefficients for fixed effects, *Z* is the design matrix for the random effects, *u* is the vector of the random effects, and *ε* is the vector of residuals. The BLUP values were considered as new phenotypic values in the subsequent phenotypic analysis and GWAS analysis.

Broad-sense heritability (*H^2^*) was calculated for each trait as follows:


(2) $$H^{2}=\frac{V_{g}}{V_{g}+\frac{V_{GL}}{L}+\frac{V_{e}}{L*R}}$$

where L is the number of sites; R is the number of replications; and $V_{g}$ , $V_{GL}$ , and $V_{e}$ represent the genotypic variance, the interaction between inbred lines and environmental variance, and the error variance, respectively. The analysis of heritability was conducted using the R package asreml (Butler, 2009).

### 2.4 Genome-wide association study

Genotypic data of maize association mapping panel were obtained from Maizego (www.maizego.org/Resources.html). Quality control of genotypic data of 407 inbred lines was performed, leaving 798,048 SNPs with a minor allele frequency (MAF) more than 0.05 and a call rate greater than 0.9. The GWAS for 20 ear-related traits from BJ, SY, and BLUP were conducted using GEMMA (Zhou and Stephens, 2012). GEMMA builds upon the mixed linear model (MLM) framework and leverages eigen decomposition to increase computational speed significantly. To account for population structure, the first 10 principal components calculated by principal component analysis (PCA) of the 798,048 SNPs using PLINK 1.09 software were used. GEMMA with 798,048 SNPs and the parameter “-gk 2” was employed to calculate the relative relationship of 407 inbred lines. *P*-values for SNP-trait associations were calculated using the likelihood ratio test, and Genetic Type I error calculator (Li et al., 2012) was used to establish the significant threshold for SNPs linked to traits. A suggestive *P*-value threshold of 2.50E-6 (1/effective number = 1/399313.86) was adopted to define statistical significance. Finally, Manhattan plots and quantile-quantile (QQ) plots were utilized to visualize the results. All candidate genes were annotated by annotate variation (ANNOVAR) (Wang et al., 2010) according to the maize B73 reference genome (B73 RefGen_v4) available in EnsemblPlants (http://plants.ensembl.org/Zea_mays/Info/Index) and national center for biotechnology information (NCBI) Gene database (https://www.ncbi.nlm.nih.gov/gene).

The degree of explanation of phenotypic variation (PVE) for each SNP was calculated as follows:


(3) $$\text{PVE}=\frac{2*beta^{2}*af*\left(1-af\right)}{2*beta^{2}*af*\left(1-af\right)+se^{2}*2*N*af*\left(1-af\right)}$$

where *N* is the sample size, *se* is the standard error of effect size for the genetic variant of interest, *beta* is the effect size for the genetic variant of interest, and *af* is the MAF for the genetic variant of interest.

### 2.5 Haplotype analysis and expression heatmap of candidate genes

Haplotype analysis was performed on the SNPs that were significantly associated with each trait. The Confidence Intervals method in Haploview 4.2 (Barrett et al., 2005) was utilized, allowing for the comparison of SNPs within the 95% confidence interval of D′, with each comparison being classified as “strong linkage disequilibrium,” “inconclusive,” or “strong recombination.” SNPs with over 95% of comparisons categorized as “strong linkage disequilibrium” were grouped as a block, which indicates a strong linkage among these SNPs. Haploview’s visualization tool was utilized to display the linkage disequilibrium (LD) values. All adjacent SNPs within a block showed LD values above 0.9. Finally, the phenotypic differences among different haplotypic materials were analyzed using ANOVA.

Candidate gene expression levels for different maize tissues of B73 were obtained from the MaizeGDB qTeller database (https://qteller.maizegdb.org/) and visualized using data from the Kaeppler lab (Stelpflug et al., 2016) and the Briggs lab (Walley et al., 2016) to generate heatmaps. The pheatmap package (Kolde, 2019) in the R 4.2.1 was used to generate the heatmap.

## 3 Results

### 3.1 Phenotypic extraction of maize ear

A high-throughput phenotypic measurement method and system, based on panoramic surface image, was utilized in our study to replace conventional approaches for assessing maize ear-related traits. This novel approach enabled the extraction of not only the traditional traits such as EL, row number per ear (RNE), and kernel size but also the multi-scale traits such as ear shape (ES) and color. A total of 2,333 valid image samples from 407 inbred lines were obtained, resulting in the acquisition of 20 ear-related traits. These traits were categorized into two groups on the basis of the acquisition method: seven traditional traits and 13 novel traits. The high-throughput phenotypic measurement method and system employed in this study facilitated a more efficient evaluation of phenotypic traits for future research. Detailed descriptions and definitions of the 20 ear-related traits can be found in **Table 1**.

### 3.2 Phenotypic characteristics of maize ear

**Figure 2:**
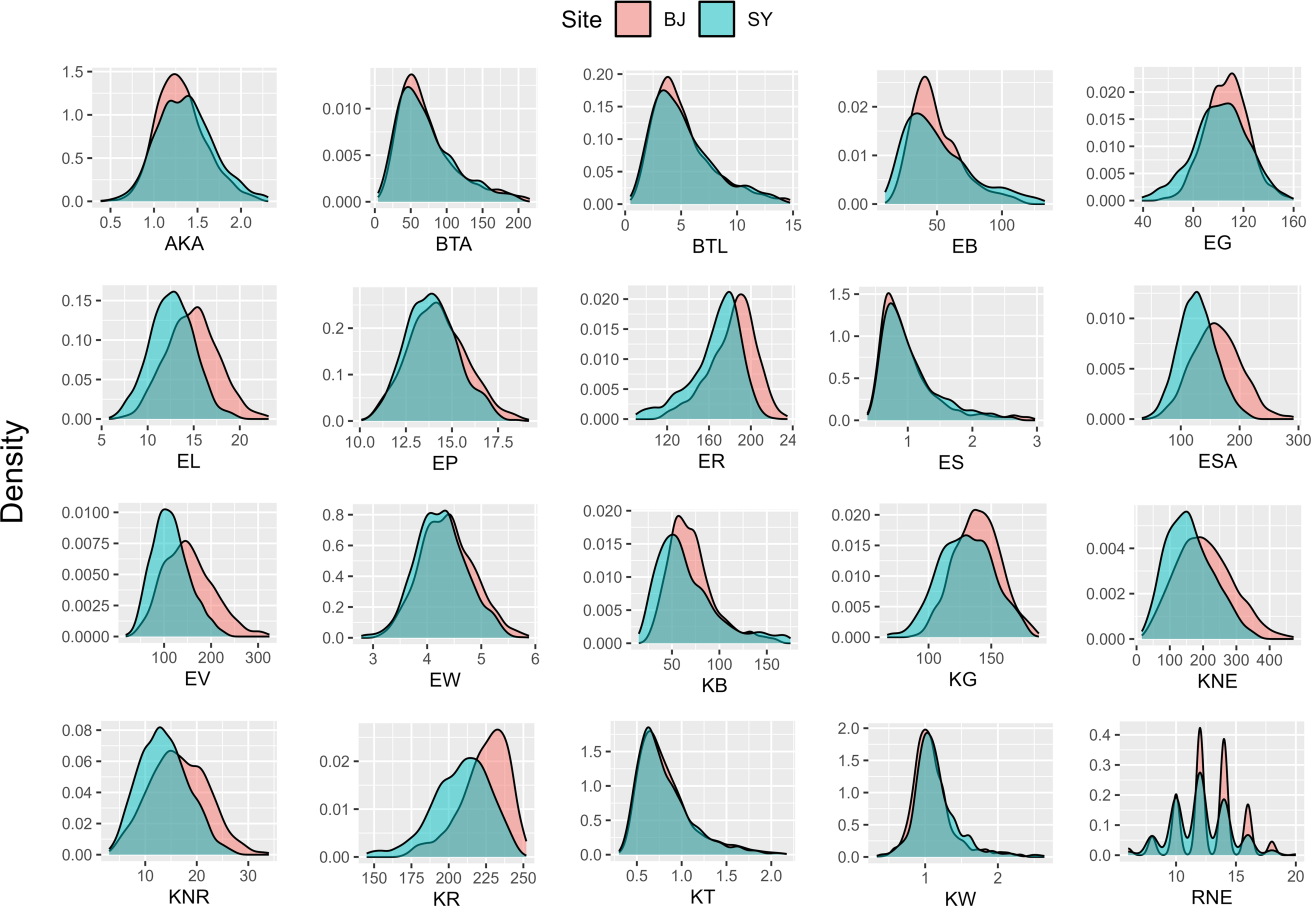
Histogram of 20 ear-related traits obtained from two sites. BJ, Beijing; SY, Sanya.

The statistical analysis of 20 ear-related traits from two sites (**Supplementary Table 1**) revealed a wide range of continuous variation among the inbred lines in the maize association analysis population. The variation coefficient spanned from 0.0718 to 0.5575, indicating significant differences in phenotypic traits. Specifically, within the BJ group, the coefficients of variation ranged from 0.0718 to 0.5572. For the SY group, the coefficients of variation fell between 0.0922 and 0.5575. Moreover, histograms of data from both BJ and SY suggested that all traits were quantitative, displaying a continuous variable distribution (**Figure 2**). These findings highlight the extensive and varied nature of phenotypic traits within the population, with practical applications for future research.

A comprehensive analysis of heritability was carried out for 20 ear-related traits using high-throughput phenotypic measures and systems. The results revealed a range of heritability values from 0.3002 to 0.8320 (**Supplementary Figure S1**). Notably, three color traits displayed high heritability values (*H^2^* > 0.82), warranting further exploration of their underlying genetic mechanisms. In contrast, both bald tip traits [barren tip length (BTL) and barren tip area (BTA)] exhibited modest heritability (0.3002 and 0.3447, respectively), suggesting that environmental factors may have a greater impact on their formation. Overall, 70% of ear traits displayed heritability greater than 0.5, indicating that genetic factors still greatly influence maize ear traits. These findings provide valuable insights into the genetic and environmental factors that contribute to phenotypic variation in maize ear traits.

### 3.3 Relationship among ear traits in maize

**Figure 3:**
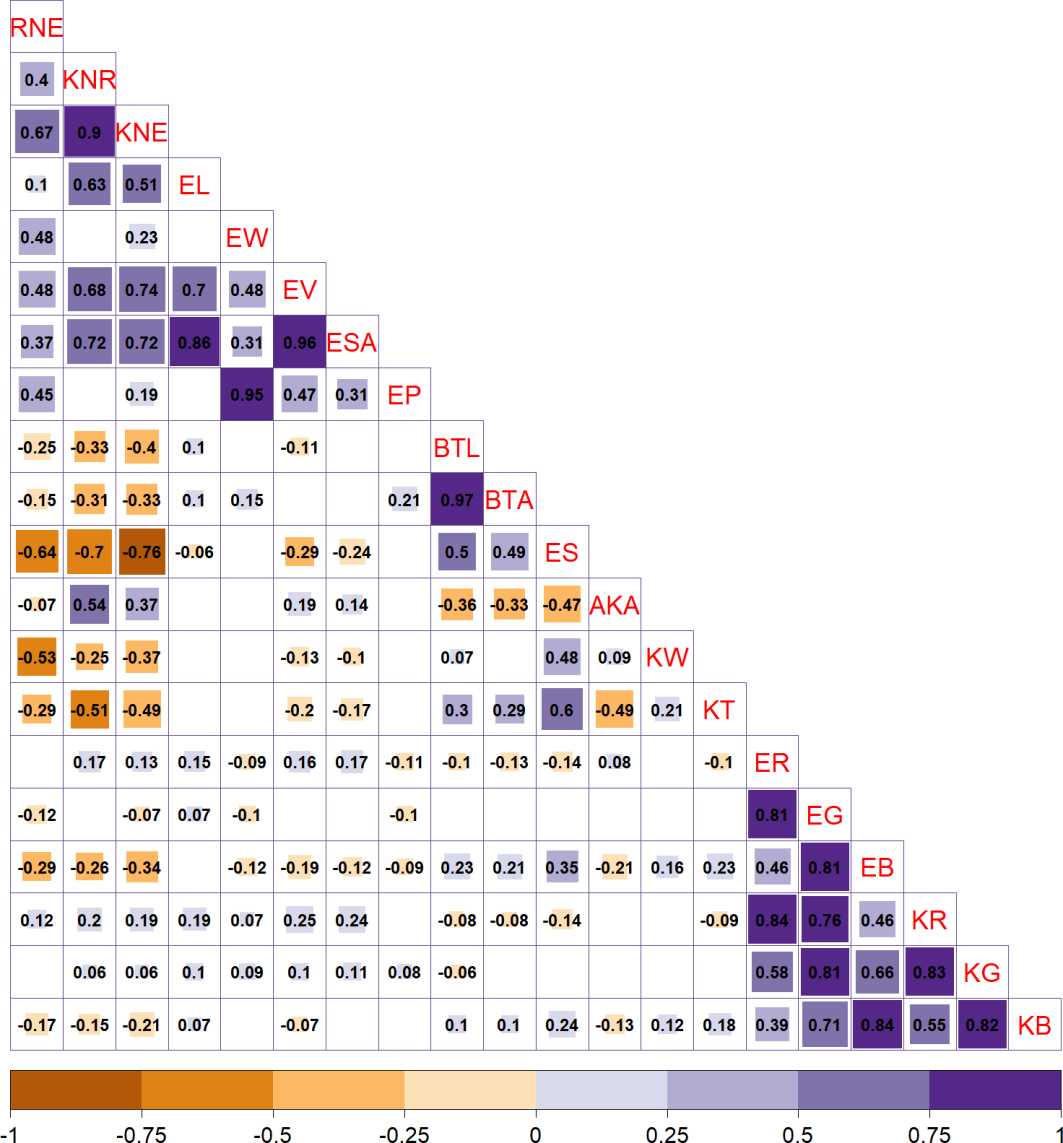
Results of correlation analysis among 20 ear-related traits. The size of the square in the upper right corner represents the significance, as per adjusted *P*-values. The number in the lower left corner represents the correlation coefficient.

The correlation between traits was determined using Spearman’s rank correlation coefficient method, and the conclusive correlation results were obtained by applying multiple testing corrections and reporting the corrected *P*-values. The analysis revealed several significant correlations among the traits. Specifically, a strong positive correlation was found between kernel number per row (KNR) and kernel number per ear (KNE) with r = 0.85 and adjusted *P* < 0.05. Furthermore, these two traits were significantly and positively correlated with EL, EV, and ear surface area (ESA) with r > 0.51 and adjust *P* < 0.05 but were significantly negatively correlated with ES and kernel thickness (KT) with r < −0.49 and adjust *P* < 0.05. In addition, the two bald tip traits (BTL and BTA) exhibited a strong positive correlation with each other with r = 0.97 and adjust *P* < 0.05. They were also found to have a significant negative correlation with average kernel area (AKA) (r > −0.33, adjust *P* < 0.05) but a significant positive correlation with ES (r < −0.49, adjust *P* < 0.05). Notably, ES, a novel trait investigated in this study, was found to be correlated with traditional traits of interest, KNR and KNE, as well as bald tip traits (BTL and BTA), highlighting its importance as a reference for the screening and determination of typical traits of maize ear. Finally, all six color traits showed a significant positive correlation with each other (r > 0.46, adjust *P* < 0.05) as shown in **Figure 3**.

### 3.4 Differences in ear traits among subpopulations

**Figure 4:**
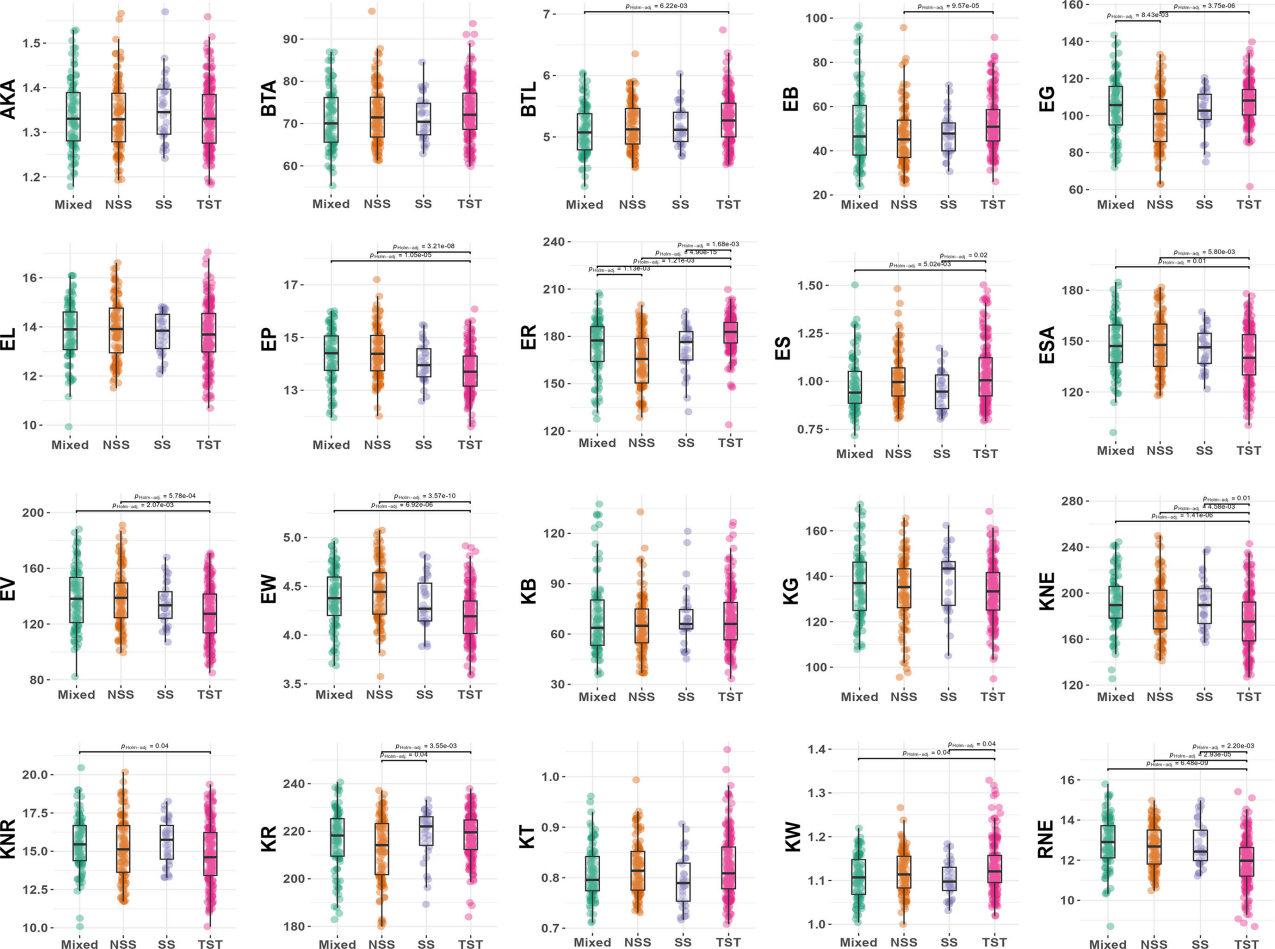
Phenotypic differences among four subpopulations. NSS, non-stiff stalk lines; SS, stiff stalk lines; TST, tropical-subtropical lines; Mixed, mixed lines.

To further analyze the differences among subpopulations, a pairwise comparison test analysis of 20 ear-related traits in maize was conducted (**Figure 4**). The results showed that the inbred lines of TST subpopulation had distinct characteristics and were significantly different from at least one subpopulation in 14 traits (70%) (adjust *P* < 0.05). Among them, RNE, KNE, and ER showed significant differences between TST and the other three subpopulations (adjust *P* < 0.05) (**Figure 3**). It indicated that the inbred lines of TST were significantly different from other subpopulations in row numbers per ear, kernel numbers per ear, and ear color, and these traits could be used as selection characteristics for maize varieties. Interestingly, ER of TST was significantly higher than that in other subpopulations, whereas KNE and RNE were significantly lower. Moreover, the distinctions between TST and NSS were more pronounced, with KR, EB, ER, and EG being significantly higher in TST than that in NSS, and EP, ESA, EV, and ear width (EW) being significantly lower in TST. It suggested that tropical materials were higher than temperate materials in color red-green-blue (RGB) channel values, and tropical materials were smaller than temperate materials in ear size and morphology.

However, AKA, BTA, EL, KT, kernel B (KB), and kernel G (KG) did not show significant differences among subpopulations, indicating that these traits alone cannot be used as selection characteristics to differentiate between tropical or subtropical materials from temperate ones in maize varieties.

### 3.5 Significant SNPs identified by GWAS

In this study, a comprehensive GWAS analysis was conducted on 20 ear-related traits, utilizing GEMMA under three scenarios: BJ, SY, and BLUP. The analysis yielded 275, 434, and 362 significant SNPs for BJ, SY, and BLUP, respectively. Of these, the number of significant SNPs detected varied from 0 to 25 for traditional traits and from 2 to 247 for novel traits in a single scenario (**Table 2**). It is noteworthy that both traditional and novel traits showed detectable SNPs. The results highlight the importance of investigating each trait because they tend to have genetic variants that may be linked to important phenotypic differences. Furthermore, the discovery of significant SNPs for novel traits allows for the possibility of exploring previously unexamined aspects of ear-related traits.

**Table 2:** Summarized statistical results of GWAS for 20 ear-related traits.

Traits	Site	SNP	Gene	Mean of PVE (%)	Range of PVE (%)
AKA	BJ	15	12	5.13	4.76–5.73
	SY	9	7	5.17	4.74–5.68
	BLUP	12	10	5.25	4.60–5.75
BTA	BJ	10	6	5.77	4.55–7.86
	SY	3	3	5.52	4.56–6.89
	BLUP	8	6	5.84	3.98–7.01
BTL	BJ	19	15	5.01	4.02–6.63
	SY	4	5	5.29	4.80–6.46
	BLUP	9	8	5.22	4.08–6.16
EB	BJ	13	17	4.87	3.78–6.97
	SY	82	31	7.82	5.1–15.69
	BLUP	62	27	7.21	5.12–12.69
EG	BJ	45	20	6.34	4.82–9.73
	SY	148	84	8.43	5.12–24.17
	BLUP	157	53	7.96	5.02–19.31
EL	BJ	7	5	4.81	4.29–5.67
	SY	7	6	5.58	5.38–5.77
	BLUP	3	2	5.32	5.16–5.48
EP	BJ	0	0	0	0
	SY	8	5	5.37	5.00–6.22
	BLUP	5	4	5.35	5.09–5.80
ER	BJ	66	22	7.23	4.48–11.97
	SY	247	95	8.75	5.11–24.09
	BLUP	197	74	8.26	5.01–20.56
ES	BJ	22	16	5.45	4.30–6.18
	SY	15	14	5.1	4.33–6.91
	BLUP	9	9	4.97	4.22–5.39
ESA	BJ	12	6	4.47	4.14–5.05
	SY	7	5	5.2	4.98–5.52
	BLUP	6	2	5.49	5.07–5.73
EV	BJ	30	11	5.01	4.19–5.61
	SY	11	11	5.04	4.33–6.22
	BLUP	9	5	5.5	5.09–6.21
EW	BJ	1	1	5.21	5.21
	SY	13	8	4.98	4.08–6.14
	BLUP	7	5	5.21	5.02–5.51
KB	BJ	19	11	5.51	4.02–7.27
	SY	12	11	5.57	5.16–6.60
	BLUP	17	12	5.96	5.03–8.44
KG	BJ	13	11	4.62	3.87–5.85
	SY	35	28	5.96	4.75–9.04
	BLUP	14	9	5.91	5.22–7.62
KNE	BJ	5	6	5.06	4.68–6.16
	SY	18	11	5.44	4.62–6.81
	BLUP	24	12	4.82	4.25–5.60
KNR	BJ	6	9	5.02	4.46–5.94
	SY	15	11	4.91	4.17–5.60
	BLUP	5	5	5.59	4.92–6.05
KR	BJ	16	12	5.17	4.60–6.71
	SY	120	44	6.44	5.03–11.71
	BLUP	105	40	6.38	6.91–11.49
KT	BJ	24	20	5.07	4.34–8.47
	SY	3	4	5.01	4.79–5.33
	BLUP	9	8	4.82	4.41–5.38
KW	BJ	13	3	6.36	5.26–11.02
	SY	2	2	5.86	5.13–6.59
	BLUP	2	3	5.87	5.82–5.91
RNE	BJ	15	9	5.07	4.10–5.77
	SY	12	3	5.51	4.56–6.13
	BLUP	25	14	5.11	4.10–6.55

BJ, Beijing; SY, Sanya; BLUP, best linear unbiased prediction; PVE, the phenotypic variance explained by SNPs.

The phenotypic interpretation of the significantly associated SNPs varied across different traits, with the PVE by all SNPs ranging from 3.78% to 24.17%. Most of the loci showed a small PVE, averaging 7.06% (**Table 2**). However, 24 significant loci for three ear color traits [ear R (ER), ear G (EG), and ear B (EB)] had PVEs of more than 15%. Notably, chr1.s_48334591 and chr1.s_48336474, both located on chromosome 1, were common to all three color traits. These findings suggest that certain SNPs can explain a substantial proportion of the phenotypic variation in ear color traits and can thus be utilized in breeding programs aimed at improving these traits.

### 3.6 Identification and annotation of candidate genes

Using the maize B73 reference genome (B73 RefGen_v4) available in EnsemblPlants, gene annotation was performed on all SNPs that displayed significant associations with 20 ear-related traits across three distinct scenarios. As a result, 174, 203, and 174 candidate genes were identified for BJ, SY, and BLUP, respectively. The number of candidate genes varied widely depending on the scenario and trait evaluated. For traditional traits, the number of candidate genes was generally modest, with a range of 0 to 15, whereas, for novel traits, there was a broader range, spanning from 2 to 95. Interestingly, our comparison of SNP results with gene annotations revealed that some genes were associated with multiple SNPs. This was particularly notable for color traits, suggesting that these genes could play a key role in regulating ear-related traits.

To further investigate the candidate genes obtained from the three scenarios, we searched for their detailed functional descriptions in NCBI Gene databases. A total of 58 candidate genes associated with ear-related traits were found to have detailed functional descriptions common to more than two scenarios (**Table 3**). Of these, 40 genes were associated with color traits, and it was observed that 82.5% (33/40) of these genes were located on chromosome 1. This finding indicates that the region in question is most likely associated with the control of ear or kernel color in maize.

**Table 3:** Candidate genes for 20 ear-related traits identified in at least two scenarios.

Gene	Description	Chromosome	Trait
*Zm00001d020416*	U-box domain-containing protein 35	7	AKA (BLUP and SY)
*Zm00001d026129*	Cyclin superfamily protein, putative	10	BTA (BJ) and BTL (BJ and BLUP)
*Zm00001d017241*	TOC1b	5	BTA (BLUP) and BTL (BJ and BLUP)
*Zm00001d023468*	VAMP protein SEC22	10	BTA (BLUP and SY) and BTL (BLUP and SY)
*Zm00001d029417*	P-loop containing nucleoside triphosphate hydrolase superfamily protein	1	EB (BJ and BLUP), EG (BJ and BLUP), KB (BJ and BLUP), and KG (BJ and BLUP)
*Zm00001d028863*	Uncharacterized protein	1	EB (BJ, BLUP, and SY), ER (BJ, BLUP, and SY), EG (BJ, BLUP, and SY), KB (SY), KG (BLUP and SY), and KR (BLUP and SY)
*Zm00001d028824*	Putative galacturonosyltransferase-like 7	1	EB (BLUP), EG (BJ, BLUP, and SY), ER (BLUP and SY), and KR (BLUP and SY)
*Zm00001d028825*	NAD(P)-binding Rossmann-fold superfamily protein	1	EB (BLUP), EG (BJ, BLUP, and SY), ER (BLUP and SY), and KR (BLUP and SY)
*Zm00001d048373*	White cap1	9	EB (BLUP) and KB (BJ and BLUP)
*Zm00001d028840*	DUF4378 domain protein	1	EB (BLUP and SY), EG (BJ, BLUP, and SY), ER (BJ, BLUP, and SY), KB (BLUP and SY), KG (BLUP and SY), and KR (BJ, BLUP, and SY)
*Zm00001d028838*	Long cell-linked locus protein	1	EB (BLUP and SY), EG (BJ, BLUP, and SY), ER (BJ, BLUP, and SY), KB (SY), KG (SY), and KR (BLUP and SY)
*Zm00001d028841*	Putative protein TPRXL	1	EB (BLUP and SY), EG (BJ, BLUP, SY), ER (BJ, BLUP, SY), KB (SY), KG (SY), KR (BLUP, SY)
*Zm00001d028829*	Calmodulin-binding protein 60 G	1	EB (BLUP and SY), EG (BJ, BLUP, and SY), ER (BJ, BLUP, and SY), KG (SY), and KR (BJ, BLUP, and SY)
*Zm00001d028834*	Peroxisomal biogenesis factor 11 family protein	1	EB (BLUP and SY), EG (BJ, BLUP, and SY), ER (BJ, BLUP, and SY), KG (SY), and KR (BJ, BLUP, and SY)
*Zm00001d028823*	F-box domain containing protein expressed	1	EB (BLUP and SY), EG (BLUP and SY), ER (BLUP and SY), KG (SY), and KR (BLUP and SY)
*Zm00001d028822*	F-box domain containing protein	1	EB (BLUP and SY), EG (BLUP and SY), ER (BLUP and SY), and KR (BLUP and SY)
*Zm00001d028771*	Ubiquitin-activating enzyme E1 3	1	EB (SY), EG (BLUP and SY), and ER (BLUP and SY)
*Zm00001d028773*	NADH dehydrogenase [ubiquinone] 1 alpha Subcomplex subunit 8-B	1	EB (SY), EG (BLUP and SY), and ER (BLUP and SY)
*Zm00001d028736*	Protein YIPF5 homolog	1	EB (SY), EG (BLUP, SY), ER (BLUP, SY), KG (BLUP and SY), and KR (BLUP and SY)
*Zm00001d028739*	Sialyltransferase-like protein 4	1	EB (SY), EG (BLUP and SY), ER (BLUP and SY), KG (SY), and KR (BLUP and SY)
*Zm00001d028733*	SNF1-related protein kinase	1	EG (BLUP and SY), ER (BLUP), KG (BLUP and SY), and KR (BLUP and SY)
*Zm00001d028735*	Isocitrate dehydrogenase pseudogene	1	EG (BLUP and SY), ER (BLUP), KG (BLUP and SY), and KR (BLUP and SY)
*Zm00001d028868*	Putative AMP-dependent synthetase and ligase superfamily protein	1	EG (BLUP and SY) and ER (BLUP and SY)
*Zm00001d028759*	Pyruvate decarboxylase 3	1	EG (BLUP and SY), ER (BLUP and SY), KG (SY), and KR (BLUP and SY)
*Zm00001d028811*	Glycosyltransferase 5	1	EG (BLUP and SY), ER (BLUP and SY), KG (SY), and KR (BLUP and SY)
*Zm00001d028769*	Ethylene-overproduction protein 1	1	EG (BLUP and SY), ER (BLUP and SY), and KR (BLUP and SY)
*Zm00001d039041*	Cell wall protein AWA1-like	6	EG (BLUP and SY), ER (BLUP and SY), and KR (BLUP and SY)
*Zm00001d028804*	U2 small nuclear ribonucleoprotein B’’	1	EG (BLUP and SY), ER (BLUP and SY), and KR (SY)
*Zm00001d028806*	Tassel seed 2	1	EG (BLUP and SY), ER (BLUP and SY), and KR (SY)
*Zm00001d028742*	Omega-3 fatty acid desaturase, chloroplastic	1	EG (BLUP and SY) and KR (BLUP and SY)
*Zm00001d028725*	Beta3-glucuronyltransferase	1	EG (SY) and ER (BJ, BLUP, and SY)
*Zm00001d028625*	DOF30 transcription factor	1	EG (SY) and ER (BLUP and SY)
*Zm00001d028812*	Programmed cell death protein 2	1	EG (SY) and ER (BLUP and SY)
*Zm00001d029461*	Protein LAZ1 homolog 2	1	EG (SY) and ER (BLUP and SY)
*Zm00001d029462*	Inter-alpha-trypsin inhibitor heavy chain-related	1	EG (SY) and ER (BLUP and SY)
*Zm00001d030129*	S-adenosyl-L-methionine-dependent methyltransferase superfamily protein	1	EG (SY) and ER (BLUP and SY)
*Zm00001d028757*	Transcription factor bHLH140	1	EG (SY), ER (BLUP and SY), and KR (BLUP and SY)
*Zm00001d028452*	Glyoxal oxidase	1	EG (SY), ER (BLUP and SY), and KR (SY)
*Zm00001d045026*	Auxin response factor 16	9	EL (BJ and BLUP)
*Zm00001d049325*	Protein aq_1857	4	EP (BLUP and SY) and EW (BLUP)
*Zm00001d051328*	Probable WRKY transcription factor 12	4	EP (BLUP and SY) and EW (BLUP and SY)
*Zm00001d051329*	Vesicle-associated protein 2-1	4	EP (BLUP and SY) and EW (BLUP and SY)
*Zm00001d028810*	Transaminase/transferase, transferring nitrogenous groups	1	ER (BJ and BLUP)
*zma-MIR159f*	MicroRNA MIR159f	3	ES (BJ and BLUP), KT (BJ), and KW (BJ)
*Zm00001d045513*	Protein binding protein	9	ES (BLUP and SY) and KNE (BLUP and SY)
*Zm00001d007100*	Pentatricopeptide repeat-containing protein At3g46790, chloroplastic	2	ESA (BLUP), EV (BLUP), KNE (BJ and BLUP), and KNR (BJ)
*Zm00001d033905*	Growth-regulating factor-interacting factor1	1	ESA(SY) and EV (BLUP and SY)
*Zm00001d048372*	Glutamyl-tRNA synthetase, cytoplasmic	9	KB (BJ and BLUP)
*Zm00001d018904*	ATP binding protein	7	KB (BLUP and SY)
*Zm00001d023292*	Trigger factor-like protein TIG, Chloroplastic	10	KB (BLUP and SY)
*Zm00001d006246*	La-related protein 1C	2	KG (BJ) and RNE (BJ and BLUP)
*Zm00001d021241*	Glucose inhibited division protein A - sarcosine oxidase pseudogene	7	KR (BLUP and SY)
*Zm00001d002360*	Embryonic protein DC-8	2	KT (BJ and BLUP)
*Zm00001d002370*	Hydroxycinnamoyltransferase 2	2	KT (BJ and BLUP)
*Zm00001d053080*	Probable receptor protein kinase	4	KW (BJ) and RNE (BJ, BLUP, and SY)
*Zm00001d012689*	Protein CURVATURE THYLAKOID 1C, chloroplastic	8	KW (BJ and BLUP)
*Zm00001d051384*	Cortical cell-delineating protein	4	RNE (BJ and BLUP)
*Zm00001d011056*	Phospholipase A1-IIgamma	8	RNE (BJ and BLUP)

BJ, Beijing; SY, Sanya; BLUP, best linear unbiased prediction.

### 3.7 Haplotype analysis of significant loci

**Figure 5:**
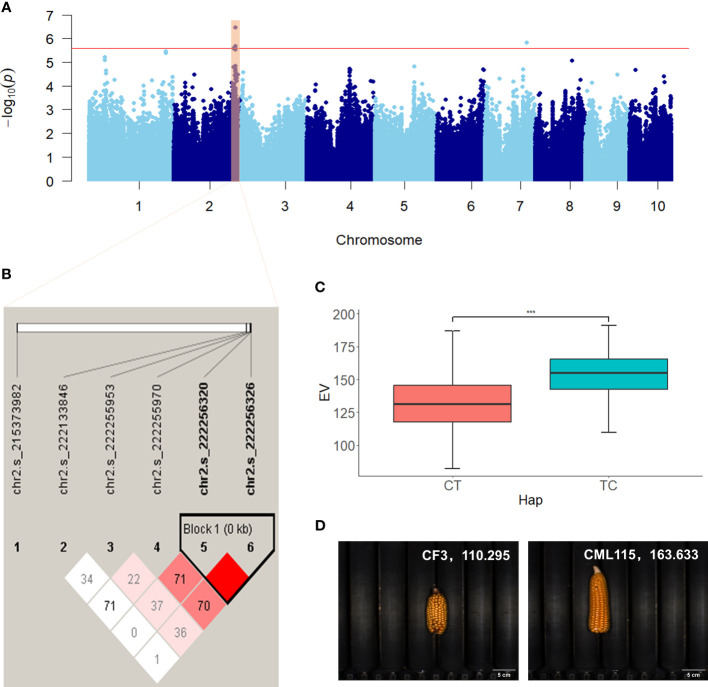
Manhattan plot and haplotype analysis results of EV. **(A)** Manhattan plot displaying the results of the GWAS for the BLUP of EV. **(B)** LD among the SNPs associated with EV, showing a haplotype block on chromosome 2. **(C)** Phenotypic differences between the haplotypes CT and TC. **(D)** Representative sample images for each haplotype. ***, adjusted P <0.001.

**Figure 6:**
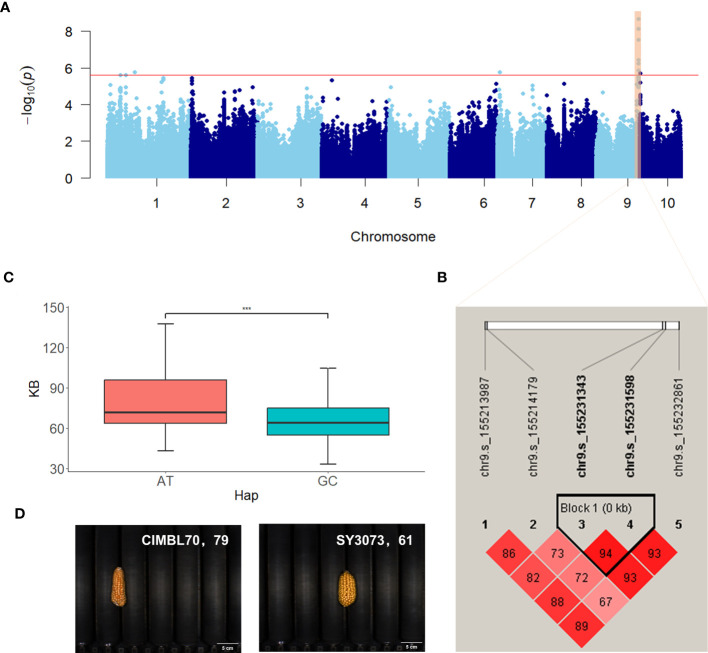
Manhattan plot and haplotype analysis results of KB. **(A)** Manhattan plot displaying the results of the GWAS for the BLUP of KB. **(B)** LD among the SNPs associated with KB, highlighting a haplotype block on chromosome 9. **(C)** Phenotypic differences between the haplotypes AT and GC. **(D)** Representative sample images for each haplotype. ***, adjusted P <0.001.

The significant SNPs associated with ear-related traits were subjected to haplotype analysis using Haploview 4.2 (Barrett et al., 2005). Inbred lines within the population exhibited varying combinations of haplotypes at multiple SNP loci, thereby revealing the genetic diversity and variation of the maize population under study. Both traditional and novel ear-related traits were studied in this study, including six novel color traits defined by RGB values. For the traditional trait EL, two SNPs located within the exon region of *Zm00001d011060* on chromosome 8 (chr8.s_137865777 and chr8.s_137865788) were identified in two scenarios (BLUP and BJ) and significantly associated with EL. These SNPs formed two haplotypes, GG and AA, with respective population frequencies of 0.762 and 0.237. Inbred lines with the AA haplotype had longer EL compared with those with the GG haplotype (**Supplementary Figure S2**). For the novel trait EV, two SNPs (chr2.s_222256320 and chr2.s_222256326) located within the exon region of *Zm00001d007100* on chromosome 2 formed two haplotypes (CT and TC), which exhibited notable differences in ear volume (EV) (**Figure 5**). The population frequencies of CT and TC were 0.935 and 0.064, respectively. Ears with the TC haplotype had a larger volume when compared with those with the CT haplotype. For the color trait KB, two SNPs (chr9.s_155231343 and chr9.s_155231598) located within the intron and exon region of *Zm00001d048372* on chromosome 9 were significantly associated with KB and identified in two scenarios (BLUP and BJ). These SNPs formed two haplotypes (AT and GC) with respective population frequencies of 0.886 and 0.101, and inbred lines with the AT haplotype had a higher KB value compared with those with the GC haplotype (**Figure 6**).

### 3.8 Expression analysis of candidate genes

**Figure 7:**
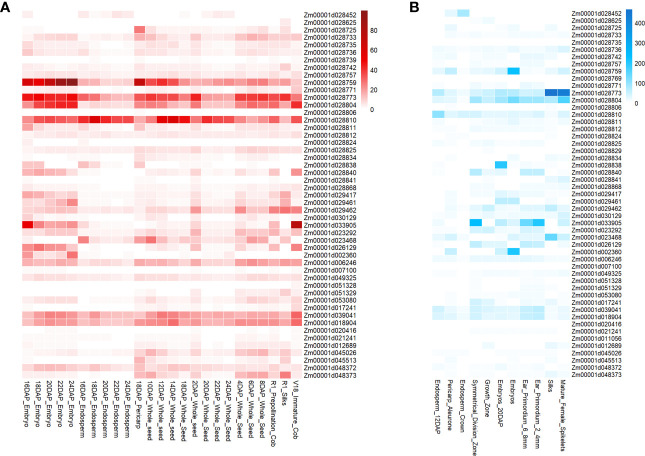
Gene expression levels of candidate genes associated with ear-related traits observed in tissues specifically related to ear development. **(A)** Data from the Kaeppler lab (Stelpflug et al., 2016). **(B)** Data from the Briggs lab (Walley et al., 2016).

In this study, we utilized maizeGDB qteller to analyze the expression of 58 candidate genes in different maize ear-related tissues or organs. It was found that nearly all of these genes were expressed in one or more tissues (expression levels exceeded 1), highlighting their potential importance in ear development (**Figure 7**). Forty-nine of the 58 genes were expressed in at least one instance in 25 previously studied tissues or organs (Stelpflug et al., 2016) (**Figure 7A**), whereas 51 of the 58 genes were expressed at least once in 11 other tissues or organs (Walley et al., 2016) (**Figure 7B**). Notably, several color-related genes exhibited the highest levels of expression in specific tissues, such as pericarp and cob. One candidate gene for EG (SY) and ER (BJ, BLUP, and SY), *Zm00001d028725*, encodes beta3-glucuronyltransferase and had the highest expression level of 26.7 observed 18 days after pollination (DAP), compared with other tissues. Another candidate gene for ER (BJ and BLUP), *Zm00001d028810*, which encodes transaminase/transferase motifs, had sustained high expression in the endosperm. Moreover, we found that candidate genes for two novel ear-related traits (ESA and EV) were highly expressed in specific tissues. For example, *Zm00001d033905*, a candidate gene for ESA(SY) and EV (BLUP, SY), encodes growth-regulating factor-interacting factor 1 and had the highest expression level observed in immature cob of V18 stage, with a value of 75.9 as compared to other tissues. Furthermore, we observed that *Zm00001d033905* had elevated levels of expression in the tip of ear primordium, indicating its potential candidacy as a candidate gene for ESA (SY) and EV (BLUP and SY).

## 4 Discussion

### 4.1 High-throughput phenotypic measurement method and system facilitates maize ear phenotypic acquisition

Maize is the primary grain crop cultivated in China due to economic and social development and the continuous reduction in arable land. An urgent need exists to further enhance maize production, with breeding and planting new varieties being among the most direct and crucial methods to achieve this. Morphological traits of maize ears are essential parameters for selecting and breeding new varieties, including EL, diameter, number of rows, number of kernels in row, and number of kernels in ear. In this study, a high-throughput panoramic image–based method and system for maize ear testing was used to obtain the ear-related traits of the 407 inbred lines. The method and system employed achieved a better balanced the efficiency and accuracy of maize ear testing, with an average efficiency of image acquisition and calculation efficiency of 15 ears/min and four ears/min, respectively. Furthermore, a calculation accuracy of 99% for EL and 98.89% for ear row number was achieved (Du et al., 2018). Because of the high throughput ear testing system, in addition to the traditional ear-related traits, novel phenotypic traits such as ear geometry, color, and texture were calculated using panoramic images that encompassed the entire kernel distribution on the ear surface. High-throughput ear phenotyping methods have been proven to be capable of replacing manual techniques, liberating individuals from laborious manual tasks. This cutting-edge technique significantly improves the efficiency of maize variety testing and provides a comprehensive dataset to support large-scale and commercial breeding programs. By employing the high-throughput phenotypic measurement method and system, maize ear phenotypic acquisition becomes streamlined. Furthermore, high-throughput ear phenotyping methods are crucial in the maize variety testing process as they enable easy, efficient, and accurate estimation of both conventional and novel ear phenotypic traits. By using this advanced measurement technique, a comprehensive and detailed profile of maize traits was obtained, which might provide valuable insights for identifying and selecting of maize varieties with superior attributes.

### 4.2 The characteristics and significance of novel traits and traditional traits of ear in maize inbred lines

The current study achieved a significant breakthrough by utilizing a high-throughput phenotypic measurement method and system to acquire a comprehensive and detailed profile of 20 traits related to maize ears, encompassing morphology, size, color, and kernel number. For clarity, the 20 traits were classified into two groups: traditional and novel. Seven of the traits (RNE, KNR, KNE, EL, EW, EP, and BTL) were traditional traits typically assessed manually in maize variety testing. The other 13 traits consisted of six color traits (ER, EG, EB, KR, KG, and KB) and seven ear and kernel morphology traits (AKA, BTA, ES, ESA, EV, KT, and KW). These traits, which could not be quantitatively evaluated using traditional methods, were identified as novel ear-related traits.

The results of the correlation analysis revealed significant correlations among different ear-related traits. In addition, the GWAS analysis indicated the existence of shared genes among these traits. For instance, the correlation coefficient between the traditional trait BTL and the novel trait BTA was exceptionally high at 0.97, and they shared three genes, namely, *Zm00001d026129*, *Zm00001d017241*, and *Zm00001d023468*. In addition, ESA and EV showed a strong correlation coefficient of 0.96, and their correlation coefficients with two traditional traits, namely, KNE and KNR, exceeded 0.68. These four traits shared two genes, namely, *Zm00001d007100* and *Zm00001d033905*. Importantly, even negatively correlated traits shared genes, which suggested the possibility of an antagonistic relationship in phenotypic expression, although they may be regulated by the same gene. For instance, the correlation coefficient between ES and KNE was −0.76, and they shared the gene *Zm00001d045513*, whereas KW and RNE had a negative correlation coefficient of −0.53 and shared the gene *Zm00001d053080*. The identification of shared genes across different phenotypes provided insights into the genetic foundation of diverse traits. These insights were crucial for understanding the genetic mechanisms underlying complex traits in maize. Although the shared genes served as a foundation for understanding the relationships among different traits, each trait possessed its own locus and gene and, therefore, maintained its individual significance. Specifically, this study revealed numerous novel traits sharing loci with traditional traits, emphasizing their potential importance in the selection and breeding of high-yield maize varieties. Moreover, these newly identified traits could be used as references for screening and determining typical ear-related traits of maize.

This study offers significant insights into the complex genetic mechanisms underlying traits in maize and establishes a foundation for future research in this field. Through the identification of novel ear-related traits, researchers can enhance their understanding of the genetic and molecular pathways involved in maize growth and development. Furthermore, the findings of this study have practical consequences for efforts in maize breeding, providing new avenues for selecting and cultivating desirable traits. Overall, this study makes a significant contribution to the field of maize genetics and is likely to inform future research in this area.

### 4.3 Candidate genes for ear-related genes

The findings of this study highlight the significance of traits obtained through the high-throughput phenotypic measurement method and system, particularly novel traits that cannot be assessed by traditional methods. The high heritability observed for several traits (**Supplementary Figure S1**), including novel ear-related traits, suggests that genetic factors play a crucial role in maize ear-related traits, providing potential for further genetic studies. The GWAS analysis identified a number of candidate genes for these traits, further strengthening the genetic basis of these traits.

For traditional traits, it was observed that there is a strong correlation (r = 0.9511, adjust *P* = 0) between EP and EW in this study. This correlation is further supported by the identification of common genes (such as *Zm00001d049325*, *Zm00001d049326*, *Zm00001d051328*, *Zm00001d051329*, and *Zm00001d005421*) through GWAS. One of the identified genes, *Zm00001d051328*, also known as *GRMZM2G377217*, was reported to play a fundamental role in maize growth and development (Hu et al., 2021). It encodes a probable WRKY transcription factor 12, involved in various biological processes. Furthermore, a search tool for recurring instances of neighbouring genes (STRING) analysis revealed its interaction with two MYB family genes, MYB109 and MYB92, which are plant transcription factors with diverse functions in biological processes. In addition, a study on the genome-wide atlas of transcription during maize development demonstrated the involvement *Zm00001d051328* in the plant ontology (PO) term “central spike of ear (PO:0006505)” and “ear inflorescence (PO:0020136)” (Sekhon et al., 2011). These findings suggest that *Zm00001d051328* may play a critical role in the development of the central spike of the ear and the ear inflorescence, thereby potentially affecting the size of maize ear. Similarly, the gene *Zm00001d011060* emerged as a candidate gene for EL. In this study, the two identified SNPs, namely, chr8.s_137865777 and chr8.s_137865788, located in the exon region of *Zm00001d011060* on chromosome 8, demonstrated significant associations with EL in both BLUP and BJ scenarios, indicating their potential functional relevance. Furthermore, maize inbred lines carrying the AA haplotype, which contains these two SNPs, exhibited longer ELs compared with those with the GG haplotype. Although detailed functional annotation for *Zm00001d011060* is currently limited, the observed phenotypic disparities strongly suggest its critical involvement in the regulation of EL. Future investigations should be conducted to unravel the molecular mechanism underlying the control of EL in maize by *Zm00001d011060*.

In addition to traditional ear-related traits, this study also focuses on novel traits, such as ES. Through GWAS, *zma-MIR159f* has been identified as a candidate gene for ES. It has demonstrated that *zma-MIR159f* is involved in encoding the transcription factors MYB33 and MYB101 (Tsuji et al., 2006), which play a role in regulating seed dormancy and germination. Furthermore, the expression of miR159, which is induced by abscisic acid (ABA) (a key regulatory factor in plant maturation and development), reduces the sensitivity of MYB genes to ABA (Phillips et al., 2007). The expression level of *zma-MIR159f* has also been observed in the maize ear primordium at 2–4 mm and in the embryo at 38 DAP (Walley et al., 2016). In Arabidopsis, miR159 and its target genes are known to be involved in important developmental processes (Millar and Gubler, 2005). In addition, overexpression of miR159 in rice has resulted in the development of deformed, sterile, and significantly smaller flowers compared with that of wild-type plants (Smoczynska and Szweykowska-Kulinska, 2016). Overall, our study suggested that *zma-MIR159f* could be a promising candidate gene for ES, providing new insights into the developmental mechanism of maize ear growth.

Furthermore, the GWAS results have found that certain traditional ear-related traits share a common genetic foundation with novel ear-related traits. This discovery has opened up new avenues for understanding the genetic architecture of these traits. For example, the gene *Zm00001d053080* (also known as *GRMZM2G032337*) has been identified as a probable receptor protein kinase gene located on chromosome 4. Remarkably, this gene has shown significant associations with KW and RNE, with a correlation coefficient of up to −0.53. In addition, gene expression analysis has indicated that *Zm00001d053080* exhibits relatively higher expression levels in the whole seed of B73 at 6 and 8 DAP compared with other tissues (**Figure 7A**). Moreover, it displays relatively higher expression levels in the 2-mm to 4-mm and 6-mm to 8-mm stages of ear primordium development compared with other tissues (**Figure 7B**). These findings strongly suggest that *Zm00001d053080* may play a pivotal role in regulating kernel width and RNE, particularly during early ear development stages.

In this study, maize ear color was not only mainly reflected by the color of the kernels but also included the color of bald tips or the exposed cob between the rows of kernels, so the traits of ear color were slightly different from the traits of kernel color. The correlation analysis revealed a significant correlation between ear color and kernel color traits, exhibiting strong associations of 0.81–0.84 between color traits derived from the same channel. In addition, the overall correlation coefficients for ear color and kernel color ranged from 0.39 to 1, further highlighting a substantial relationship between the two. Genetic analysis further demonstrated numerous overlapping, significantly related SNPs and candidate genes between these color traits. For example, the gene *Zm00001d028840*, also known as *GRMZM2G017536*, has emerged as a strong candidate gene for color traits in this study. This gene encodes a DUF4378 domain protein and is situated on chromosome 1. Its significance in relation to color traits is highlighted by its identification as a candidate gene for EB, EG, ER, KB, KG, and KR traits in maize. In addition to this, the high significance of the SNPs associated with this gene further strengthens its candidacy for color traits. The identification of this gene provides valuable insights into the genetic basis of color traits in maize, paving the way for further research to understand its mechanism of action and potential applications in improving color quality and yield of maize crops.

Notably, *Zm00001d048373*, also known as *white cap1*, *CCD*, *wc1*, *CCD1*, and *GRMZM2G057243*, was identified as a candidate gene for EB (BLUP) and KB (BJ and BLUP) in our study. The selection for yellow- and white-kernel types has been central to postdomestication improvement of maize. It has previously been reported (Dutta et al., 2021) that the copy number of *ccd1* gene varied among different maize varieties, with yellow maize typically having one to six copies and white maize having 7 to 35 copies, whereas their wild relatives contain one to four copies of the ccd1 gene per genome. Another study (Tan et al., 2017) demonstrated that the Wc locus contained multiple, tandem copies of a carotenoid cleavage dioxygenase 1 (*Ccd1*) gene that encodes a carotenoid-degrading enzyme, playing a role in evolution of kernel color in maize. These findings suggested that *Zm00001d048373* could be an important candidate gene for the color trait in maize, specifically EB and KB. In contrast to previous research that primarily focused on classifying yellow and white maize kernels, our study took a more detailed approach by specifically examining the single color channel of the kernel in the RGB color model. By isolating and analyzing this specific color channel, we were able to gain deeper insights into the genetic factors that influence the precise coloration of maize kernels. This finer-grained investigation is of significant importance for understanding the underlying mechanisms that govern maize kernel color development, thus providing valuable insights for future breeding and improvement strategies.

It has been reported that a major effect quantitative trait locus (QTL) (qEL1) with significant association to maize EL was identified on chromosome 1 through GWAS (Luo et al., 2022a). Four candidate genes within the qEL1 interval were subsequently validated through knockout using clustered regularly interspaced short palindromic repeats (CRISPR/Cas9) technology. The results showed that the functional gene for qEL1 was *Zm00001d028915* (also known as *YIGE1*), which positively regulates maize EL. In this study, this gene was identified as a candidate gene for EG (SY) and ER (SY), and the phenotype analysis displayed a significant positive correlation between EL and EG, as well as ER after multiple verifications (**Figure 3**). The above findings suggest that there is also a certain degree of genetic correlation among these traits, potentially regulated by the same genetic mechanism.

This study emphasizes the significance of investigating novel phenotypic traits in maize through the utilization of high-throughput phenotypic measurements. The genetic loci identified in this research make a substantial contribution to our understanding of the traits associated with maize ears and enhance current genetic studies on maize ears.

## Data availability statement

The original contributions presented in the study are included in the article/**Supplementary Material**. Further inquiries can be directed to the corresponding authors.

## Author contributions

CZ and XG contributed to the study conception and design; JW and SZ analyzed the data, interpreted the results, and drafted the manuscript; XG and YZ revised the manuscript; XL, JD, CW, and WW performed field experiments and obtained phenotypic data. All authors contributed to the article and approved the submitted version.

## Supplementary material

Supplementary Figure 1 Heritability of 20 ear-related traits. These traits could be divided into two categories: Traditional traits (BTL, EL, EP, EW, KNE, KNR and RNE), and novel traits (AKA, BTA, EB, EG, ER, ES, ESA, EV, KB, KG, KR, KT and KW).

Supplementary Figure 2 Manhattan plot and haplotype analysis results of EL. **(A)** Manhattan plot displaying the results of the GWAS for the BLUP of EL. **(B)** LD among the SNPs associated with EL, highlighting a haplotype block on chromosome 8. **(C)** Phenotypic differences between the haplotypes AA and GG. **(D)** Representative sample images for each haplotype.

Supplementary Table 1 Descriptive statistics analysis of 20 maize ear-related traits. 1)RNE, Row Number per Ear; KNR, Kernel Number per Row; KNE, Kernel Number per Ear; EL, Ear Length; EW, Ear Width; EV, Ear Volum; ESA, Ear SurfaceArea; EP, Ear Perimeter; BTL, BarrenTip Length; BTA, BarrenTip Area; AKA, Average Kernel Area; ES, Ear Shape; KW, Kernel Width; KT, Kernel Thickness; ER, Ear R; EG, Ear G; EB, Ear B; KR, Kernel R; KG, Kernel G; KB, Kernel B. 2)BJ, Beijing; SY, Sanya; BLUP, best linear unbiased prediction.
