# Supplementary figures and images for: Investigating the genetic basis of maize ear characteristics: a comprehensive genome-wide study utilizing high-throughput phenotypic measurement method and system
Source: Front Plant Sci. 2023 Aug 28;14:1248446. doi: 10.3389/fpls.2023.1248446 (PMC10493325; doi:10.3389/fpls.2023.1248446)

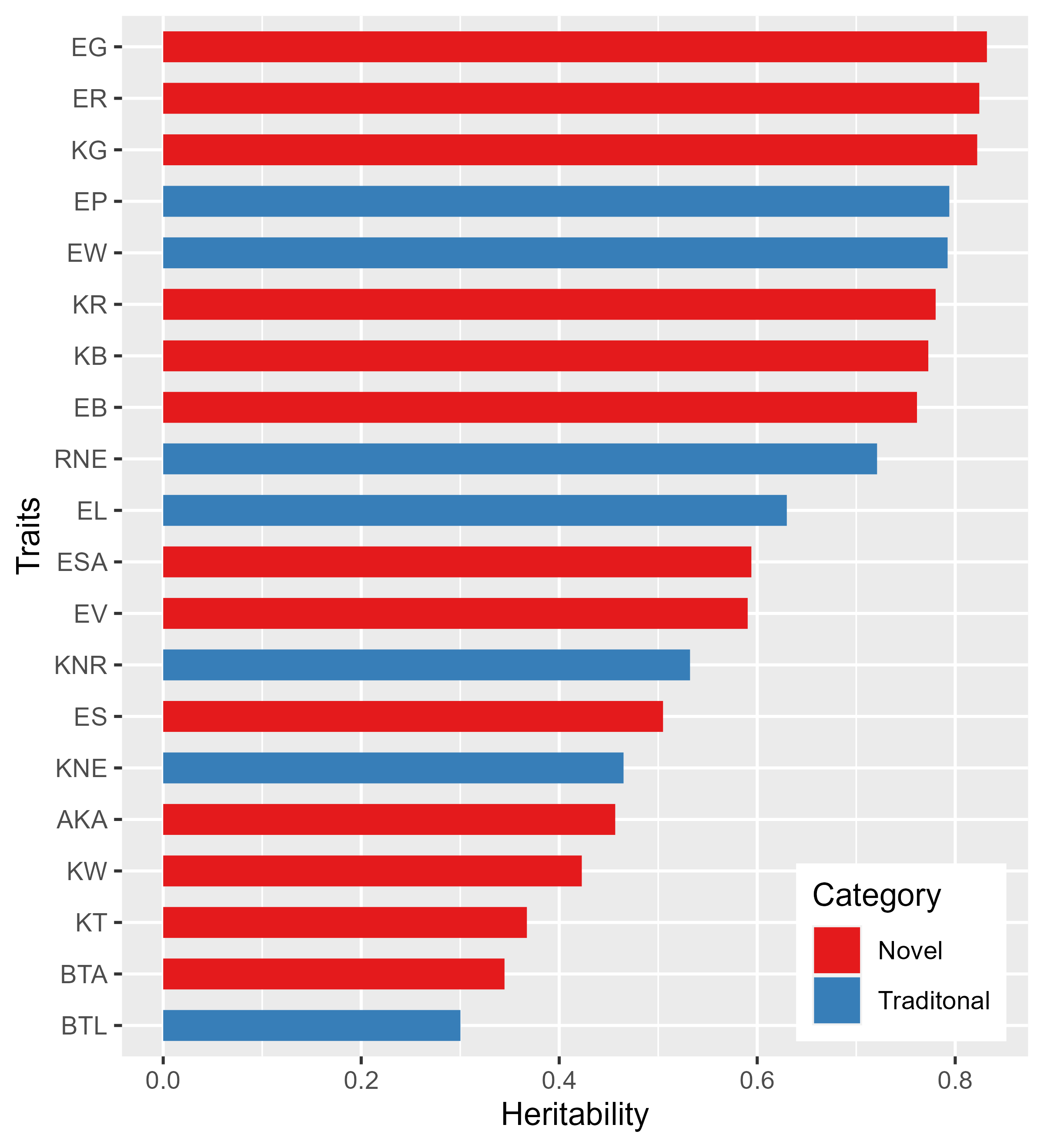

Supplement: Supplementary Figure 1 — Heritability of 20 ear-related traits. These traits could be divided into two categories: Traditional traits (BTL, EL, EP, EW, KNE, KNR and RNE), and novel traits (AKA, BTA, EB, EG, ER, ES, ESA, EV, KB, KG, KR, KT and KW). [file Image_1.jpeg]

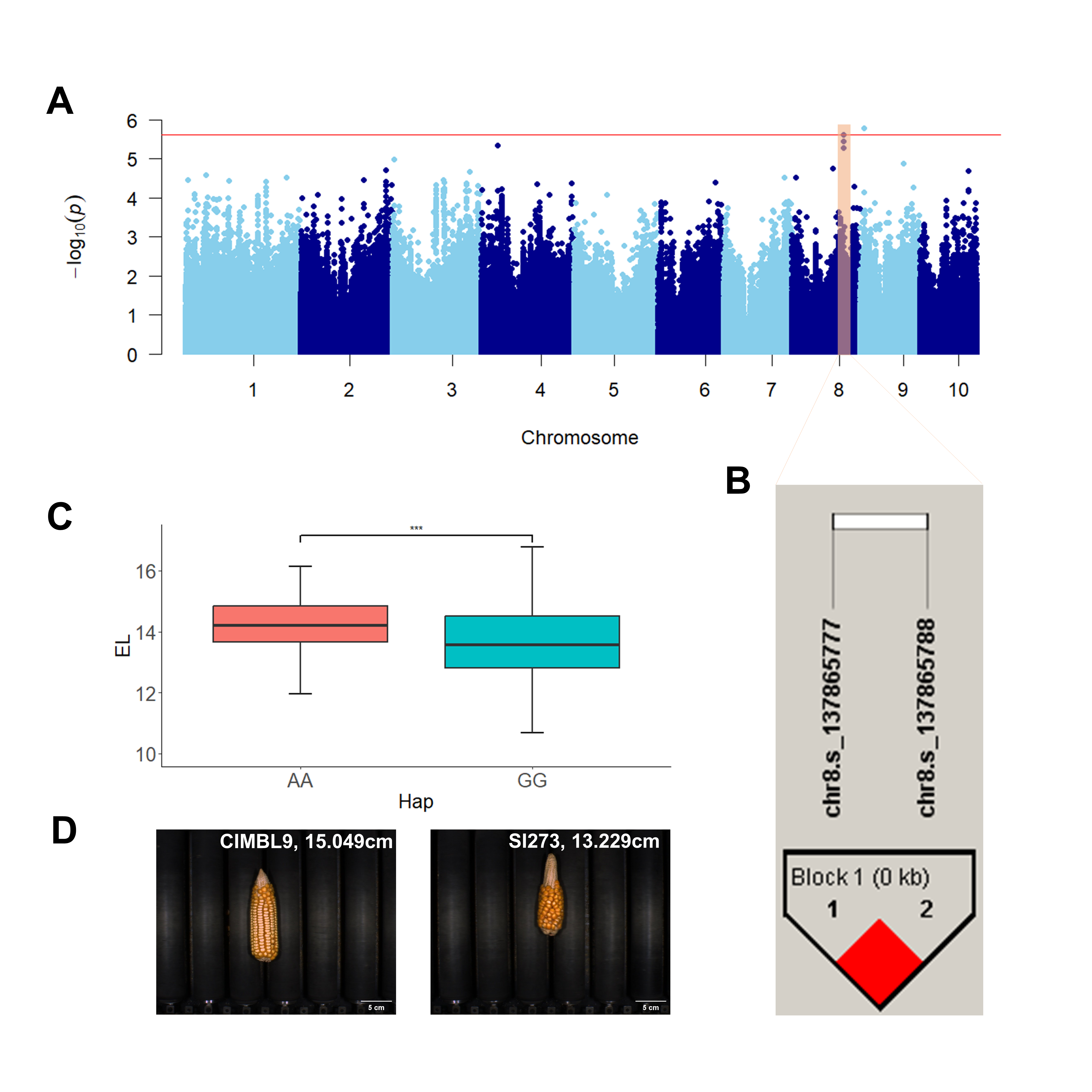

Supplement: Supplementary Figure 2 — Manhattan plot and haplotype analysis results of EL. (A) Manhattan plot displaying the results of the GWAS for the BLUP of EL. (B) LD among the SNPs associated with EL, highlighting a haplotype block on chromosome 8. (C) Phenotypic differences between the haplotypes AA and GG. (D) Representative sample images for each haplotype. [file Image_2.jpeg]
